# A systematic review on physical mutagens in rice breeding in Southeast Asia

**DOI:** 10.7717/peerj.15682

**Published:** 2023-10-18

**Authors:** Rosina Baadu, Khim Phin Chong, Jualang Azlan Gansau, Muhammad Rawi Mohamed Zin, Jedol Dayou

**Affiliations:** 1 Biotechnology Programme, Faculty of Science and Natural Resources, Universiti Malaysia Sabah, Kota Kinabalu, Sabah, Malaysia; 2 Malaysian Nuclear Agency, Selangor, Malaysia; 3 Energy, Vibration and Sound Research Group (e-VIBS), Faculty of Science and Natural Resources, Universiti Malaysia Sabah, Sabah, Malaysia

**Keywords:** Physical mutagens, Induced mutations, Mutation breeding, Rice cultivation

## Abstract

In the 1920s, Lewis Stadler initiated the introduction of permanent improvements to the genetic makeup of irradiated plants. Since then, studies related to breeding mutations have grown, as efforts have been made to expand and improve crop productivity and quality. Stadler’s discovery began with x-rays on corn and barley and later extended to the use of gamma-rays, thermal, and fast neutrons in crops. Radiation has since been shown to be an effective and unique method for increasing the genetic variability of species, including rice. Numerous systematic reviews have been conducted on the impact of physical mutagens on the production and grain quality of rice in Southeast Asia. However, the existing literature still lacks information on the type of radiation used, the rice planting materials used, the dosage of physical mutagens, and the differences in mutated characteristics. Therefore, this article aims to review existing literature on the use of physical mutagens in rice crops in Southeast Asian countries. Guided by the PRISMA Statement review method, 28 primary studies were identified through a systematic review of the Scopus, Science Direct, Emerald Insight, Multidisciplinary Digital Publishing, and MDPI journal databases published between 2016 and 2020. The results show that 96% of the articles used seeds as planting materials, and 80% of the articles focused on gamma-rays as a source of physical mutagens. The optimal dosage of gamma-rays applied was around 100 to 250 Gy to improve plant development, abiotic stress, biochemical properties, and nutritional and industrial quality of rice.

## Introduction

Climate change poses a significant threat to national agricultural production, with climate change fingerprints causing 76 droughts, floods, temperature anomalies, and storms in the last 5 years (Watts et al., 2021). Rice, being one of the world’s most essential crops, is particularly vulnerable to climate change impacts, including sea-level rise, floods, salinity, increased carbon dioxide, higher temperatures, water shortages, and pests, diseases, and weeds. This is indeed the main threat to food security, especially in countries of Southeast Asia. About 162 million hectares were harvested worldwide in 2019, of which 85.2% were in Asia (138 million hectares) and 43 million hectares (26.5% of the global rice harvested) were in Southeast Asia alone (FAOSTAT, 2021). Parallel to this, Southeast Asia’s domestic rice consumption has risen from 103,595,000 metric tonnes in 2016/17 to 103,966,000 metric tonnes in March 2020/21 (USDA, 2021). As rice is the primary source of energy in Southeast Asia, the decline in food production due to climate change is a severe threat to food security.

To mitigate this issue, it is necessary to develop new rice varieties that can adapt to climate change. One such breeding technique is mutation breeding, which uses physical or chemical mutagens to generate new forms of valuable agricultural production. There are approximately 852 varieties of rice mutants publicly released to farmers (IAEA, 2021) contributing to agricultural improvement between 1950 to 2021 *via* physical and chemical mutagenesis or somaclonal variation. Total of 81.3% (693) of the total rice mutant develop using physical mutagens. The number of rice mutant varieties in the countries of Southeast Asia is shown in Table 1. Southeast Asia accounts for 10% of total rice mutants used physical mutagens. Most of the physical mutagen sources used are gamma-rays with doses of 50 Gy up to 450 Gy. The most frequently used gamma-ray dose is 200 Gy in the development of new variants. Only 4% mutant rice are derived from fast neutron sources (10 and 20 Gy), and a mixture of gamma-rays and neutrons.

**Table 1:** Number of rice mutant varieties by physical mutagen treatment in the countries of Southeast Asia (1950–2021).

Country	Number of varieties	Country	Number of varieties
Indonesia	28	Thailand	7
Vietnam	21	Myanmar	6
Philippines	11	Malaysia	2
Source: IAEA (2021).			

Mutation breeding was introduced to obtain the highest mutagenesis efficiency in terms of a maximum number of targeted mutations while conserving plant variety and a low background mutation rate. Few studies have been clarified that several factors influence by the effect of irradiation including the radiation aspect in terms of mutagen dosage, source of mutagen, and duration of exposure (Jan et al., 2012), and the plant characteristics such as stage of development, and tissue architecture, and genome organization. With this knowledge, the plant breeder will be able to regulate and limit additional cost that may occur in manpower, time, field area, and the rounds of crossing/backcrossing required to clean up undesired background mutations (Spencer-Lopes et al., 2018).

Historically, the concept of radiation-induced mutagenesis for crop improvement dates back to the late 20^th^ century which was introduced by Stadler (Oladosu et al., 2016; Beyaz & Yildiz, 2017). Lewis John Stadler of the University of Missouri, whose initial research was focused on field plot technique and agronomy, later transitioned into genetics and concentrated on the effects of X-ray treatment in maize and barley. Stadler’s work served as the foundation for another type of plant breeding known as mutation breeding. Since then, mutation breeding has involved the creation of new varieties through the generation and utilization of genetic variability *via* chemical and physical mutagenesis. Furthermore, the state of knowledge on the efficient use of physical/chemical mutagens (JAERI, 2001), the use of *in vitro* mutation techniques, such somaclonal variation in improving fruits (Predieri, 2001), and the role of induced mutation in crop improvement (Babagana et al., 2017), are all summarized in a number of conference proceedings, research papers, and reviews. Surprisingly, there is still a dearth on the use of physical mutagens in rice production and rice quality, particularly in Southeast Asia. The output of physical mutagen in rice breeding might be of significant relevance for plant breeders dealing with improvement and development of new cultivars.

The present review article analyzes the existing literature on physical irradiation used in rice breeding in Southeast Asia in particular.

## Impact and mode of action of important physical mutagens on plants

There are numerous physical mutagens that can have an effect on plants, and each has a different mode of action. Generally, plants’ DNA structure is altered by physical mutagens, resulting in genetic variations that can produce desirable traits.

Gamma-rays can have both positive and negative effects on plants, depending on the dose and genotype. Gamma-rays, for example, can ionize atoms in the DNA molecule, causing breaks in the DNA strands or changes in the base sequences. According to Efendi et al. (2017), gamma-rays have a stronger effect on the surface of plants cells because short-wavelength photons gamma rays are stronger than photons of visible light. Plants that have been irradiated with gamma-rays may germinate faster as a result. Furthermore, gamma-ray-induced mutations are known to be the most effective in a wide range of mutations because gamma-rays penetrate deeply into target tissues, are less destructive, and can produce a broader spectrum of mutations raging from SNPs and small indels to deletions greater than one million base pairs (Gurushidze et al., 2017; Ramchander et al., 2022).

The effect of the ion beam on plants was determined by several factors including the type of the ions, the accelerator used, the dose of ion radiation, and the plant’s species and stage of development. Double-strand breaks are produced by ion beam irradiation and exhibit a broad spectrum and high frequency. It is because, as reported in the case of *Arabidopsis*, ion beam irradiation can cause half of the mutants to have point-line mutations and the other half to have large DNA alterations such as translocations, inversions, and large deletions (Abe et al., 2007). In terms of mode of action on plants, electron beam is similar to ion-beam radiation but it is thought to be less precise method.

Neutrons, like gamma-rays, are a type of ionizing radiation. Neutrons are not electromagnetic radiation like gamma-rays, but are uncharged particles. When neutrons interact with matter, they can knock atomic nuclei out of their normal positions, causing ionization and excitation of the atoms. According to Aliyu, Aliyu & Adamu (2017), neutrons cause mutations by breaking chemical bonds in the DNA molecule, deleting or substituting a nucleotide, or both. As a result, the radiation should be applied at the appropriate dose, which is determined by the radiation intensity and duration of exposure.

Physical mutagen has an ability to generate a wide range of genetic variability and this can be used as important tool in plant breeding. This variability can be used to develop new plant varieties with desirable characteristics such as disease resistance or improved yield. A few rice varieties have been developed as part of a current study. IR64, the most widely grown in South and Southeast Asia with many positive agronomic characteristics including high yield potential, wide adaptability, tolerance to multiple diseases and pests, and good eating quality, was one of the rice mutants induced by physical mutagen and released in Southeast Asia (Wu et al., 2005). M-202 was next, a mutant variety of IR64 with improved grain quality and resistance to disease that was confirmed to carry the DGWG *sd-1* allele and develop in Thailand (Jia & Jia, 2022). In 1997, the hybrid PSB Rc72H (*Mestizo*) develop tolerance to biotic and abiotic stress after being exposed to Cobalt-60. After a while, PSB RC82 with improved grain quality and tolerance to abiotic stress, as well as a mutant variety of PSB Rc72 was released in 2000. This variety was widely used after it was released because it was high yielding and had good grain quality, similar to IR64 (Launio et al., 2008). Meanwhile, in Malaysia, the Malaysia Agricultural Research and Development Institute (MARDI) developed and release MR219, a modern rice variety with improved grain quality and yield in January 2001 (Oladosu et al., 2014). Finally, in Thailand, RD6 variety was developed for drought tolerance (Bunnag & Suwanagul, 2017). The number of rice varieties induced by physical mutagens is expected to increase as the technology further develops and is adopted in the region.

## Methodology

This review adhered to the PRISMA Statement (Preferred Reporting Items for Systematic Review and Meta-analysis) as a guide to retrieve relevant articles on physical mutagens in rice cultivation. Relevant articles were assessed from a specific perspective before being selected as the primary references for exploring the physical mutagens used in rice production in Southeast Asian countries.

To conduct the review, publicly available databases such as Scopus, Science Direct, Emerald Insight, and MDPI journal databases were searched. A systematic review was performed using Mesh terms including “induced mutation”, “physical mutagen”, “physical agent”, “mutagen”, “ionizing radiation”, “radiation”, “mutation breeding”, “rice”, “paddy”, “*Oryza sativa*”, “rice cultivation” alone or in combination with “OR” and/or “AND”.

The PRISMA Statement, which comprises a 27-item checklist and a four-flow diagram, was utilized as a guide to retrieve relevant articles on physical mutagens in rice cultivation (Moher et al., 2009). The review process involved four main steps, namely identification, screening, eligibility, and inclusion. Table 2 outlines the criteria used to determine article eligibility and exclusion. Firstly, the review focused on articles published within the past 5 years (between 2016 and 2020) in selected journals, as these sources provide up-to-date and current findings, processes, and best practices. Secondly, only articles published in English were included to prevent any misunderstanding or complications that may arise from translation. Thirdly, the countries of Southeast Asia share similar cultural values, climates, technologies, industrial sectors, social and urban development, and are located in the same geographical area. Thus, articles that focus on these countries were selected for the review. Lastly, only articles with empirical data were included, while book series, conference proceedings, article reviews, and trade publications were excluded.

**Table 2:** The criteria for inclusion and exclusion in the review.

Criterion	Eligibility	Exclusion
Date/timeline	Between 2016 and 2020	<2016
Language	English	Non-English
Countries	Southeast Asian	Non-Southeast Asian
Source type	Journal (research articles)	Journal (review articles), book series, conference proceedings, a trade publication

**Figure 1:**
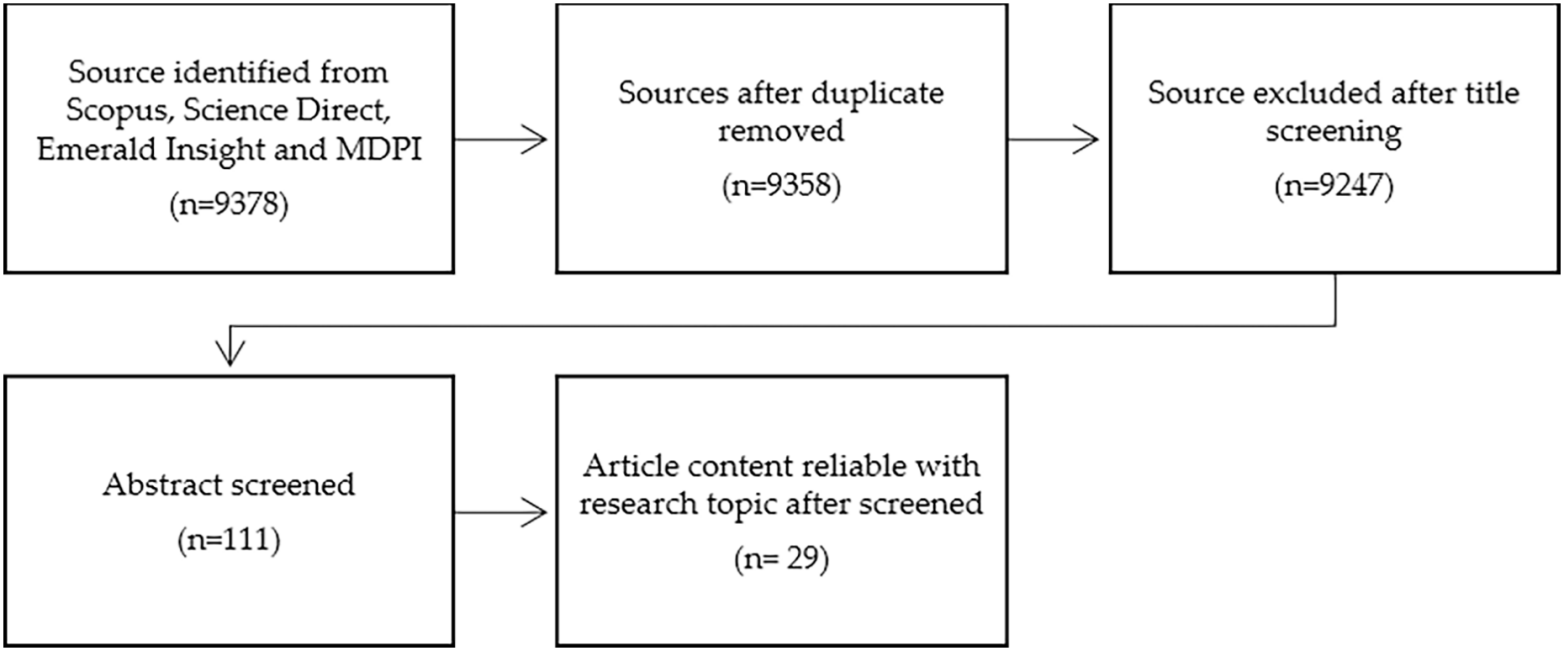
The flow chart of the analysis (modified from Moher et al., 2009).

The systematic review process was carried out in a progressive manner, starting with the identification stage, followed by screening, eligibility, and inclusion (as shown in Fig. 1). During the identification process, the aforementioned key phrases were used in the literature search. In the screening stage, the obtained results were checked for duplicate articles, and any duplicates were removed from the data collection. Out of the 9,378 articles that were initially screened, 20 articles were excluded due to various reasons, including being published before 2016 or after 2020, being published in a non-English language, not focusing on Southeast Asian countries, and not being research articles. In the eligibility process, 9,247 articles were assessed for the full article, and 9,136 articles were excluded after the screening process. The reasons for exclusion were that some articles did not focus on rice production, did not use physical mutagens as irradiation agents, and were conducted in countries other than Southeast Asia. In the end, 28 articles were used for the quantitative analysis.

After going through the systematic review steps, the remaining journal articles were carefully assessed and analyzed. The assessment began with the abstract and then proceeded to the full articles. Data was extracted and analyzed to design an appropriate theme of data. A quantitative approach was undertaken using a systematic review to address the research question on the physical radiation types used in rice cultivars in Southeast Asian countries. To organize the sub-theme around the main themes, a typology framework was used.

## Result and discussions

**Figure 2:**
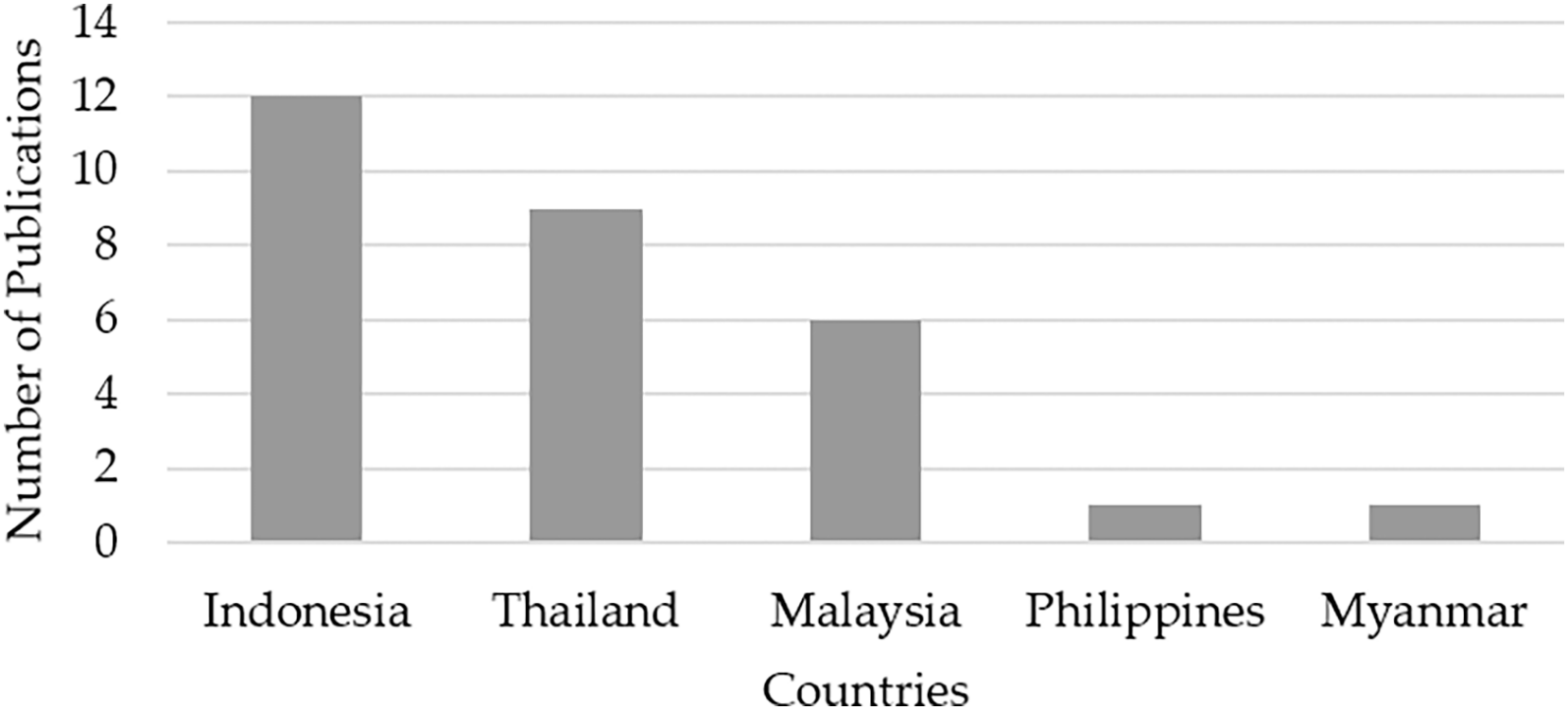
The numbers of articles on the use of physical mutagens in rice cultivation published in Southeast Asia from 2016 to 2020.

A total of 28 papers meeting the review criteria were identified, with the distribution of their origin given in Fig. 2. It can be observed that Indonesia had the highest number of relevant papers (12), followed by Thailand (eight), Malaysia (six), and the Philippines and Myanmar published two papers each. All the papers are listed in Table 3 based on their country of origin for clarity. These papers were analyzed from various aspects including the rice cultivar, plant material, mutagen used, optimum dosage found, and any altered rice qualities resulting from the mutagenic treatment.

**Table 3:** The list of papers complies with the selection criteria used in the literature review.

Authors	Country	Rice cultivar	Plant material	Mutagen	Dosage	Mutated characters
Herwibawa, Sakhidin & Dwi Haryanto (2019)	Indonesia	Inpago Unsoed 1 (aromatic upland)	Seed	Gamma ray	100 Gy	Plant development and abiotic stress
		Rojolele (aromatic lowland)			150 Gy	Plant development
		Inpari 13 (non-aromatic lowland)			150 Gy	Plant development and abiotic stress
		Cirata (non-aromatic upland)			150 Gy	
Pratiwi et al. (2020)		Mentik Wangi			150 Gy	Plant development
					200 Gy	
					250 Gy	
Sri Suliartini et al. (2020)		G10 Line			200 Gy	
		G16 Line				
		Baas Selem				
		Inpago Unram-1			500 Gy	
Efendi et al. (2017)		Inpari 10			250 Gy	Plant development and abiotic stress
		IR 64				
		Unsyiah-1 Simeulu				
		Unsyiah-3 Sanberasi				
		Unsyiah-5 Sibahak				
Amsal & Ishak-Ishak (2018)		Mira-1			25 & 50 Gy	Abiotic stress
Purwanto et al. (2019)		Cempo Ireng		Gamma ray (Cobalt-60)	100 Gy 200 Gy	Plant development Biochemical properties
		Cempo Melik			300 Gy	Biochemical properties
		Melik			100 Gy	Plant development
Efendi et al. (2017)		Sigupai			250 Gy	
		Cantek Manis			500 Gy	
		Rom Mokot			500 Gy	
		Ramos Mirah			250 Gy	
		Sanbei			500 Gy	
Kurniawati, Chaniago & Suliansyah (2019)		Sigah (Brown rice)			200 Gy	
Masruroh et al. (2016)		Ciherang			200 Gy	Plant development and nutritional quality
		Cempo Ireng				Plant development
Ishak (2016)		Sintanur		Gamma ray (Caesium-137)	100 Gy	Biochemical properties
Sjahril et al. (2018)		Pare Ambok		Ion beam (Argon)	10 Gy	Plant development
		Pare Lea				
Yunita et al. (2020)		Situpatenggang	Callus	Gamma ray	24.68 Gy	Abiotic stress
		Batutegi			22.15 Gy	
Meesook, Pongtongkam & Poeaim (2018)	Thailand	Riceberry rice	Seed	Gamma ray (Caesium-137)	28 Krad	Abiotic stress
Sansenya et al. (2019)		KDML 105			20, 40, 60, 80, 100, 150, 300 & 500 Gy	Biochemical properties
Techarang et al. (2018)		RD6		Ion-beam	4 × 10^16^ ions/cm^2^ (50 kV)	Plant development and biochemical properties
		Sangyod			4 × 10^16^ ions/cm^2^ (30 kV)	
Promnart et al. (2017)		RD31		Electron beam	440 Gy	Abiotic stress
Semsang et al. (2018)		KDML 105		Low-energy ion beam	150-kV	Plant development and Industry quality
Kongmon et al. (2020)		KDML 105		Ion-beam (N^+^ + N_2_^+^)	2 × 10^16^ ions/cm^2^ (60 kV)	Biochemical properties
Techarang et al. (2019)		Sangyod Phatthalung		Ion beam (N^+^)	4 × 10^16^ ions/cm^2^ (50 kV)	Plant development and biochemical properties
Malumpong et al. (2019)		JHN		Fast Neutron	33 Gy	Abiotic stress
Che Mohd Zain et al. (2016)	Malaysia	MRQ74	Seed	Gamma ray	350 Gy	Abiotic stress
Ahmad Zainuri et al. (2020)		Rice flour		Gamma ray (Cobalt-60)	8 kGy	Industrial quality
Kadhimi et al. (2016a)		MR269	Seed	Gamma ray (Caesium-137)	350 Gy	Abiotic stress
Kadhimi et al. (2016b)		MRQ74			100 Gy	Plant development
		MR269				
Yasmine et al. (2019)		Binadhan-8			162 Gy	
		NMR-152			286 Gy	
		Pongsu Seribu-2			67 Gy	
Hasan et al. (2020)		MR284			100 Gy	
Tapic et al. (2016)	Philippines	NSIC Rc144	Seed	Gamma ray	250 Gy	Plant development
Aung, Khai & Minn, 2016	Myanmar	Ayarmin	Seed	Gamma ray (Cobalt-60)	300 Gy 400 Gy	Plant development
					350 Gy	Biochemical properties

### Types of radiations used in rice mutation breeding for varietal development

**Figure 3:**
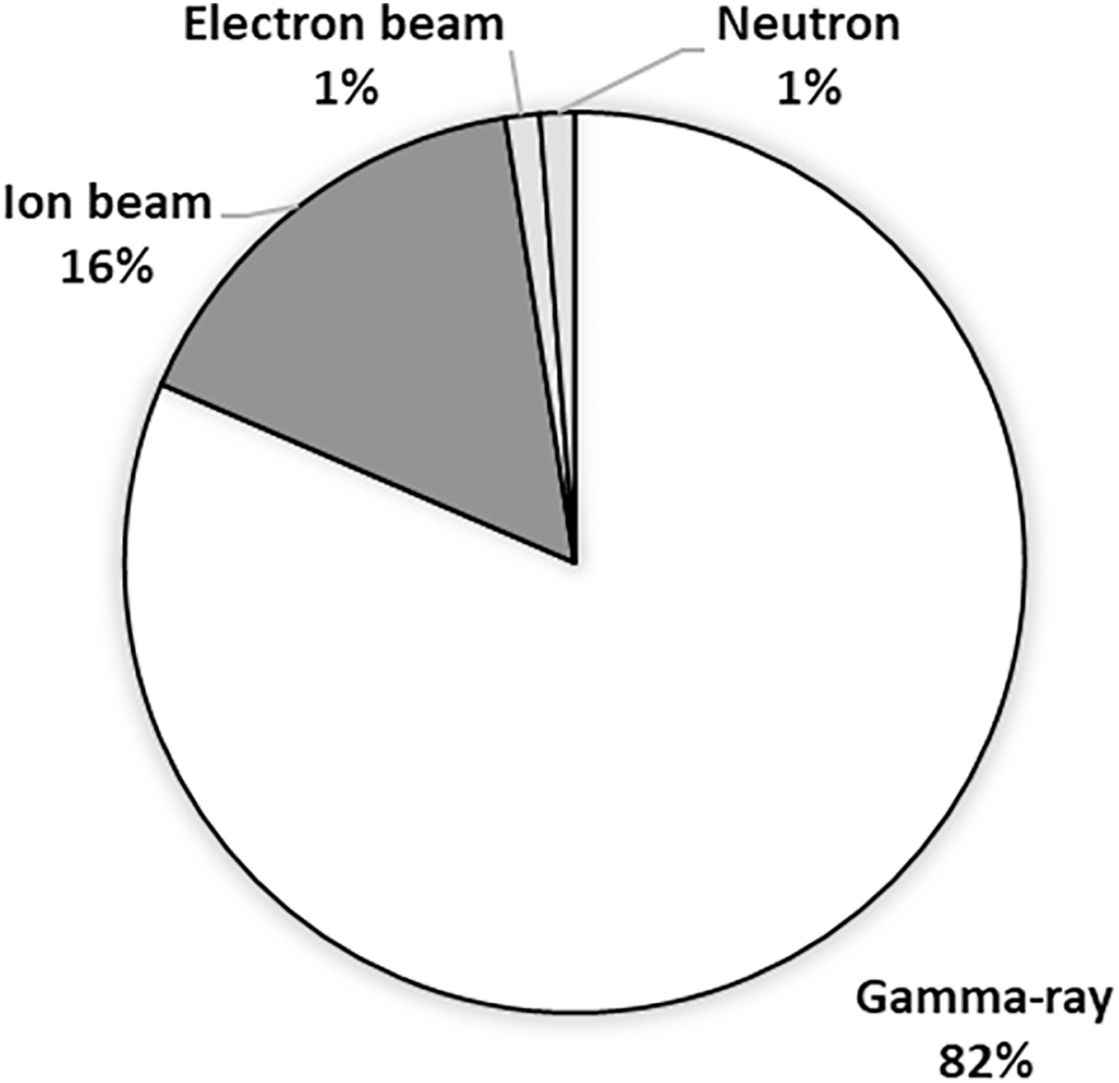
The number of developed rice varieties by individual physical mutagens in Southeast Asia from 2016 to 2020.

Physical mutagens are generated by nuclear radiation and radioactive sources, such as ultraviolet light (a non-ionizing radiation) and ionizing radiations (such as gamma-rays, x-rays, alpha and beta particles, protons, and neutrons). In Southeast Asia, physical mutagens have been used in rice production to create new cultivars, improve genetic traits, and increase yield potential. The following section provides more details on the specific physical reagents that have been used. Figure 3 illustrates the number of rice varieties developed by each physical mutagen, with gamma rays resulting in 66 rice varieties, ion beams producing 13 rice varieties, and electron beams and neutrons resulting in one rice variety each.

#### Gamma-rays

Gamma rays are electromagnetic waves with the highest energy and shorter wavelengths that can penetrate deep into biological matter. Analysis of the studies reviewed revealed that gamma rays were used as a source of physical mutagens in 21 out of 28 studies to enhance crop traits. Of these, 13 studies used Cobalt-60, and seven studies used Cesium-137 as the gamma-ray source. The radiation source was not specified in eight of the studies. Cobalt-60, which has a half-life of 5.27 years, is produced by irradiating the stable isotope Cobalt-59 with neutrons in a nuclear reactor (Roberts, 2003). On the other hand, caesium-137 has the longest half-life of 30.1 years, making it the most long-lived isotope (Parsons, 2012). According to Oladosu et al. (2016), gamma rays offer an advantage in terms of accuracy and repeatability as a physical mutagen due to their homogeneous penetration capacity in tissue.

Indonesia has developed the most varieties of rice over the last five (5) years, utilizing 28 varieties of rice, namely Inpago Unsoed 1, Rojolele, Inpari 13, Cirata, Cempo Ireng, Cempo Melik, Melik, Sigupai, Cantek Manis, Rom Mokot, Ramos Mirah, Sanbei, Ciherang, Mentik Wangi, G10 Line, G16 Line, Baas Selem, Inpago Unram-1, Situpatenggang, Batutegi, Sintanur, Sigah, Mira-1, Inpari 10, IR64, Unsyiah-1 Simeulu, Unsyiah-3 Sanberasi, and Unsyiah-5 Sibahak. In contrast to genetic improvement, a physical mutagen is also used in food production for example in Malaysia, a study by Ahmad Zainuri et al. (2020) utilized gamma-rays to enhance the industrial quality of rice flour.

#### Ion-beam

An ion beam is a charged particle beam that contains an ion. Along with gamma-rays, ion beams have been widely exploited as novel mutagens in mutation breeding programs for the production of new genetic resources in plants, with the increasing number of mutants in the database (Zengliang, 2006; Satoh & Oono, 2019). In Takasaki, Japan, the National Institutes for Quantum and Radiological Science and Technology (QST), has been pursuing fundamental research on the induction of mutation in plants and microorganisms using ion beams accelerated by an azimuthally varying field (AVF) cyclotron at Ion Accelerators for Advanced Radiation Application (TIARA) since the early 1990s. Ion beams have a high linear energy (LET) (about 10–1,000 keV/mm or higher) and cause DNA damage/alterations at high densities, such as clustered damage involving large deletions, translocations, and inversions (Souframanien et al., 2020). One of the most important reasons for the use of ion implantation mutation is that it combines the variables of mass, charge, and energy, and causes damage to biological materials (including genetic components) that primarily leads to the displacement, recombination, and compounding of biological molecules and atoms.

Recent inquire shows there are five (5) studies that utilized ion beams in improving rice traits and consists of four (4) from Thailand and one (1) from Indonesia. In Thailand, the rice varieties KDML-105 and Sangyod were utilized in addition to RD6, whilst, in Indonesia, Pare Ambok, and Pare Lea were used. The seeds were blasted with various ion beams including mixed molecular (N^+^), atomic nitrogen ions (N_2_^+^), argon, and carbon under-voltage potential of 30 and 50 kV to an ion fluence of around 1 × 10^16^ to 4 ×10^16^ ions/cm^2^ (the bombarding time was about 2 min for the fluence of 10^16^ ions/cm^2^). The results showed that in the preliminary test, the optimum conditions to achieve the survival rates of ion bombarded rice seeds from 50% to 70% was the combination of 30 kV and 1,2 and 4 × 10^16^ ions/cm^2^ fluences or 50 kV and 1 × 10^16^ ions/cm^2^.

#### Electron beam

In contrast to ion beams, alpha particles, and neutrons, which have a high linear energy transfer (LET), electron beams, gamma-rays, and X-rays are classified as low-LET radiation (Hwang et al., 2014). Low-LET radiation is sparsely ionizing, and multiple exposures may be required to adequately irradiate target tissues. According to Spencer-Lopes et al. (2018), the use of X-rays, gamma-rays, neutrons, and electron beams has proven to be extremely beneficial in medicine, biology, and industry.

Only one study from Thailand, conducted by Promnart et al. (2017), has employed an electron beam to improve the RD31 (Pathum-Thani 31) rice variety. RD31 seeds were exposed to a 440 Gy electron beam before being tested for submergence tolerance. The findings revealed the identification of 317 submergence-tolerant lines (named Sub-1 gene) from a pool of around 3,000 M3 lines.

#### Neutron

Neutrons have the strongest ionization power among the physical mutagen agents. Neutrons are high-speed nuclear particles with a remarkable penetration power on materials. Neutrons can travel great distances in air and require very thick hydrogen-containing materials (such as concrete or water) to stop them. Fortunately, neutron radiation occurs predominantly within a nuclear reactor, where feet of water provide sufficient protection. The neutron, stable within the perimeters of the atom nucleus, once released will decays with a mean lifetime of approximately 15 min, releasing various kinetic energy. Table 4 shows different neutrons in terms of energy released that are the most used for plant mutation induction.

**Table 4:** Classification of neutrons based on its energy.

Category	Energy released
Cold neutrons	<0.003 eV
Slow (thermal) neutrons	0.003–0.4 eV
Slow (epithermal) neutrons	0.4–100 eV
Intermediate neutrons	100 eV–200 keV
Fast Neutrons	200 keV–10 MeV
High energy (relativistic) neutrons	>10 MeV
Source: L’Annunziata (2016)	

In Thailand, Malumpong et al. (2019) used a 33 Gy fast neutron (FN) to induce JHN wild type and screen for salt tolerance with 150 mM sodium chloride (NaCl). M1145 lines mutant from JHN was shown to be salinity tolerant in the study, although the authors emphasized that these lines must be tested in the field to confirm the potential for actual salt tolerance.

Several physical mutagens, including gamma-rays, ion beams, and fast neutrons have been explored in this study in terms of their efficient research for rice production enhancement. Gamma rays revealed the most significant mutagens utilized to increase rice plant growth, biochemical characteristics, industrial quality, and abiotic stress. Even though neutrons are potent mutagens, fast neutrons were less popular in rice development techniques due to the significant cost involved.

### Plant materials used for irradiations with physical mutagens

In mutation treatment, the plant material used should be relatively selective to ensure consistency and success in its genetic modification, particularly its mutation characteristics. This section explores the plant materials utilized in the great invention of rice mutant breeding in more detail.

Overall, 96% (27 articles) of the reviewed articles in this study involve seeds (containing seed embryos) as the material of choice, which is most typically used as an explant for mutation induction. In general, every part of the rice plant, including dried seeds, dormant seeds (whole plants), pollen (vegetative organs), cuttings, tissue or cells *in vitro* such as leaf and stem explants, calli, cell culture, ovules, protoplasts, *etc*., has been employed in irradiation research (L’Annunziata, 2016; Spencer-Lopes et al., 2018). However, in Southeast Asian countries, seeds are the most preferred plant material used in many mutation studies because they can be stored for long periods in air-tight, refrigerated and vacuum-sealed conditions, are easy to handle, and can be transported over long distances. In addition, seeds can be irradiated in various conditions, such as dry, wet, heated, or frozen before treatment. The time required for irradiation with physical mutagens will also depend on the plant materials utilized: wet seeds, *in vitro* tissues, and plantlets may only require a few seconds of irradiation, but dry seeds may require a few minutes (Brunner, 1995).

In Thailand, researchers investigated the effects of gamma-ray irradiation on riceberry rice seeds. The study found that dry seeds exposed to 28 krad of gamma rays resulted in a survival rate of 50% (LD_50_), while exposure to 40 krad caused changes in the rice plant’s color from green to brown (Meesook, Pongtongkam & Poeaim, 2018). Similarly, Sansenya et al. (2019) studied the effects of a combination of 20 Gy of gamma rays and 20 mM of salt concentration (sodium chloride; NaCl) on the germination of KDML 105 fragrant-rice cultivars. The researchers found that the proline content increased significantly during germination, from 10.04 µg/g on day one (1) to 28.88 µg/g on day five (5) compared to 12.5 µg/g at day 5 in the absence of NaCl. Proline content is an amino acid that plays a role in plant differentiation and growth; during seed germination, the soluble protein is degraded to free amino acids by proteolytic enzymes.

Furthermore, (Techarang et al., 2018, 2019) studied the optimal conditions for ion beam irradiation of RD6 and non-aromatic Sangyod Phatthalung rice seeds. The researchers found that a combination of 50 kV accelerated mixed nitrogen ions and fluences of 4 × 10^16^ ions/cm^2^ resulted in a survival rate of 50% to 70%. Mixed N+ +N^2+^ ions have two energies, which allows lower-energy particles to sputter and scratch more surfaces, making it easier for higher-energy particles to penetrate deeply. This property allows low-energy heavy ions to interact with deeply buried DNA inside the seed embryo cells.

To make this review, a comparison was made on the type of plant materials used in irradiation by most countries and is shown in Table 5. It should be noted here that the selection of plant materials type may have a variety of impacts on the outcomes of physical mutagen-induced mutagenesis. Irradiation of pollen for example has a significant advantage because according to Singson-Asuncion (1988), it can survive in extreme experimental conditions without interfering with their function of transporting and transmitting male genetic contributions to eggs. Furthermore, chimeras in M1 plants can be minimized since irradiated pollen is delivered at the blooming stage rather than seed irradiation. Nonetheless, the utilization of pollen is frequently constrained by the difficulty of getting adequate materials from some species, as well as the short life cycle, which only lasts a few minutes. To deal with the difficulties, effective handling strategies such as storing the spikes on a Petri dish and using mature rice pollen as a source of plant materials is suggested as described by Wang et al. (2009).

**Table 5:** Rice mutation breeding plant materials.

Target plant materials	Variety/cultivar of rice	References
Seed	2^nd^ generation of rice cultivar Nipponbare	Zheng et al. (2020)
	Mentik Susu (M3)	Rachmawati et al. (2019)
	Giza 171, Giza 159, E. Yasmin, GZ6296, GZ9399 and SP70	Abo-Youssef et al. (2018)
	HC62.2	Hong et al. (2015)
	R173 *Oryza sativa* L. ssp. indica	Yang et al. (2019)
	MR284	Hasan et al. (2020)
	*Oryza sativa* L. cultivar Nipponbare	Li et al. (2016)
	MR219	Oladosu et al. (2014)
	Mira-1	Amsal & Ishak-Ishak (2018)
	Binadhan-8, NMR-152 and Pongsu Seribu-2	Yasmine et al. (2019)
	Dongan (*O. sativa L. japonica*)	Hwang et al. (2016)
	Japonica rice line DS552	Li et al. (2018)
	Taraori Basmati and Pusa Basmati 1	Singh (2006)
	Kuku Belang	Nur Aziliana et al. (2015)
Pollen	Basmati and Bellemont	Chin & Gordon (1989)
	Azucena	Singson-Asuncion (1988)
	Jiahezaozhan and Jiafuzhan	Wang et al. (2009)
Pollen and Spikelet	ADT 37 and ADT 45	Gowthami, Vanniarajan & Souframanien (2016)
Embryogenic callus	Situpatenggang and Batutegi	Yunita et al. (2020)
	Sadamota, Kachamota, Moulata and Dudhkalam	Islam et al. (2020)

The use of plant organ, tissue, and cell culture in rice genetic improvement is no longer debatable. These techniques allow for simple and rapid dissociation of chimeras, rapid and mass propagation of any target mother plants and any mutant population, *in vitro* screening and mutagenesis, and simple methods for analyzing morphogenetic mutations and physiologic changes associated with mutation induction. However, there are some drawbacks of using *in vitro* culture as an explant for rice breeding using physical mutagens technique, such as the need for a proper sterile environment and growing medium, the selection of materials must be free of abnormalities that may lead to new plants being infected, and even though the success rate is high, there is still a chance that the process stimulates a secondary metabolic chemical reaction, and the new explants or stunted cell growth, or even die off. Therefore, *in vitro* cultured cells were less used for rice breeding using physical mutagens as described by Oladosu et al. (2016).

### Optimum dosage of physical mutagens for mutation induction

Inducing genetic variation through mutagenic agents is highly dependent on the source and dose of application Radiation dose is a unit of measurement to quantify the radiation exposure. In the radiation safety principle, there are three types of doses considered—absorbed, equivalent, and effective dose. The absorbed dose is defined as the energy per unit mass deposited in an absorbing medium from the radiation, whereas the equivalent dosage is a measurement of an average absorbed dose in tissues and organs, and the effective dose is given by the sum of all irradiated tissue. The absorbed dose is measured in milligrays (mGy), whereas the equivalent and effective doses are measured in millisieverts (mSv).

The optimal dosage used in rice breeding with different sources of physical mutagen with encouraging results is assessed and discussed in this article. Mutagen dose in irradiation studies may result in different outcomes depending on the purposes, radiation safety standards, and issues involving the radiation protection of humans. In general, the higher the irradiation dose applied, the greater the possibility of mutant formation, as well as the greater the degree of destruction will arise.

The dosage applied in gamma-ray treatment ranges from 20 to 500 Gy, whereas, ion beams are around 30 to 50 kV, electron beam (440 Gy), and neutron (33 Gy). The same trend can be seen in countries other than Southeast Asia, such as Sri Lanka and India, where the optimum dosage range for gamma-ray (200–350 Gy), electron beam (290–330 Gy), X-ray (200–250 Gy), and proton beam (150–200 Gy) was discovered (Wijesena, Bentota & Nawarathne, 2019; Sao et al., 2020). Gamma rays are electromagnetic radiation that is generated by radioisotopes and in nuclear reactors. Yamaguchi et al. (2003) found that gamma-ray irradiation was a promising treatment in mutation breeding, with survival rates ranging from 40% to 60%. Gamma rays have a shorter wavelength and higher energy than protons and x-rays and can penetrate deeper into tissues, thus, making gamma rays often selected as a source of radiation compared to the others (Oladosu et al., 2016). Physical mutagens absorb at variable rates, which is referred to as the dose-effect relationship. The higher the dose applied, the lower the germination capacity, survival rate, growth performance, *etc*. On the other hand, lower doses induced less chromosomal damage and fewer negative side effects that are related to small and large insertions, deletions, and genomic rearrangements. As an example, the effect of overdose application of ion beam (high LET) may lead to the creation of aneuploids has been discussed in detail by Zengliang (2006). As a result, dose adjustment may be performed to enhance the allele types necessary for particular reverse genetics research.

In this respect, data from Table 3 showed several physical mutagens have been compiled in this study to be helpful for the efficient exploration of technological developments, particularly in rice production. However, the success of every mutagen in plant breeding is determined not only by its mutagenic effectiveness but also by its mutagenic efficiency. The rate of point mutations compared to dosage is referred to as mutagenic effectiveness, whereas efficiency is typically regarded as a measure of damage. Thus, two plants may be equally mutagenic effective because, at a given dose, both generate the same frequency of mutations. When they differ in their capacity to induce unwanted alteration such as sterility and mortality, they are said to differ in mutagenic efficiency. In rice, the ideal dosage was found to be around 100 to 250 Gy for gamma rays, 30 kV up to 150 kV for ion beam, 440 Gy for an electron beam, and 33 Gy for Fast neutrons.

### Variations/mutated characteristics generated by physical mutagens

The genetic heterogeneity caused by induced mutation has enhanced current plant breeding. During the 2016–2020 study period, researchers concentrated on improving many useful traits affecting plant size, flowering and harvesting period, abiotic tolerance, nutritional quality (composition and content of protein, oil, minerals, vitamins, *etc*.), yield variation and including industrial quality (aroma, softness and physical appearance of the grains including uniformity, whiteness, and slenderness). Overall, irradiation treatment applied to rice varieties has a positive influence at the appropriate dose. However, it is important to exercise caution when selecting mutant genotypes obtained from plant material to ensure their reliability. This precautionary is necessary due to the substantial genetic variability observed in higher generations, for example resulting from the irradiation of mature pollen with gamma-ray, as noted by Wang et al. (2009), which extends up to H_15_.

#### Plant development

Plant development refers to the processes through which plant structures emerge and mature as plants grow. Any alteration in growth pattern will directly impact maturity and productivity. It was found that the majority of the researchers used a variety of indicators to determine plant development performance. Plant height, number of tillers, number of seed/panicles, 1,000 seed weight, and flowering and harvesting period are among the most frequently used assessment as shown in Table 6. Purwanto et al. (2019) found that irradiated rice seeds with 100 Gy (Cempo Ireng; 134.8 cm and Melik; 137 cm) and 200 Gy (Cempo Melik; 135.6 cm) can produce higher plant height than controls by around 2.3–6.9%. Similarly, Herwibawa, Sakhidin & Dwi Haryanto (2019) discovered that the plant height of Inpago Unsoed 1 (121.75 cm) and Inpari 13 (113.56 cm) increased. However, the treatment was a combination of gamma radiation 100 Gy and immersion in sodium azide (SA) for 2 and 6 h, respectively.

**Table 6:** Effect of physical mutagen on the plant development characteristics in rice cultivation.

Author	Variety/dose	PH (cm)	No of tiller	1000 seed	Flowering period (DAS)	Harvesting period (DAS)
Herwibawa, Sakhidin & Dwi Haryanto (2019)	100 Gy					
	Inpago Unsoed 1	63.65	6.1	9.28	n.a	n.a
	150 Gy					
	Rojolele	50.39	4.2	4.83	n.a	n.a
	Inpari 13	75.84	7.1	15.32	n.a	n.a
	Cirata	64.81	5.5	13.99	n.a	n.a
Sri Suliartini et al. (2020)	200 Gy					
	G10 Line	19.55	n.a	n.a	n.a	n.a
	G16 Line	24.07	n.a	n.a	n.a	n.a
	Baas Selem	20.73	n.a	n.a	n.a	n.a
	500 Gy					
	Inpago Unram-1	23.65	n.a	n.a	n.a	n.a
Efendi et al. (2017)	250 Gy					
	Inpari 10	71.91	n.a	n.a	n.a	Early 6 days
	IR 64	68.29	n.a	n.a	n.a	Early 9 days
	Unsyiah-1 Simeulu	71.77	n.a	n.a	n.a	Late 1 day
	Unsyiah-3 Sanberasi	77.25	n.a	n.a	n.a	Late 22 days
	Unsyiah-5 Sibahak	74.77	n.a	n.a	n.a	Late 10 days
Purwanto et al. (2019)	100 Gy					
	Melik	137.0	25.3 (19)	n.a	Early 2 days	Early 2 days
	200 Gy					
	Cempo Melik	135.6	23.7 (19)	n.a	Late 1 day	Early 2 days
	300 Gy					
	Cempo Ireng	134.8	24.3 (17)	n.a	Late 13 days	Late 13 days
Kurniawati, Chaniago & Suliansyah (2019)	200 Gy					
	Sigah	90.98	25.88	20.66	Late 7 days	Late 2 days
Masruroh et al. (2016)	200 Gy					
	Ciherang	85.22	n.a	25.91	Early 3 days	Early 3 days
	Cempo Ireng	136.56	n.a	24.42	Early 11 days	Early 11 days
Kadhimi et al. (2016a)	100Gy					
	MRQ74	23	n.a	n.a	n.a	n.a
	MR269	22	n.a	n.a	n.a	n.a
Yasmine et al. (2019)	67 Gy					
	Pongsu Seribu-2	123.47	11.00	50	n.a	n.a
	162 Gy					
	Binadhan-8	88.63	9.66	57	n.a	n.a
	286 Gy					
	NMR-152	99.17	10.33	55	n.a	n.a

**Note:**

() = No. of the productive tiller.

Meanwhile, in an ion beam study (Techarang et al., 2018), plant height M2 mutants from the RD6 rice cultivar ranged from 103 to 135 cm, whereas M4 mutants ranged from 112 to 143 cm. M2 mutants from the Sangyod cultivar have heights ranging from 110 to 179 cm, while M4 mutants have a height ranging from 120 to 130 cm. Both RD6 and Sangyod mutants were shorter than wild cultivars, measuring 152 and 196 cm, respectively. OSRD6-4 and OSRD6-20 were selected from RD6, and OSSY-14, OSSY-17, and OSSY-23 have selected mutants from Sangyod based on the phenotypic variations that could provide higher crop yield including shorter stature, higher tillering capacity, and panicles per plant. Among the selected mutants, OSSY-23 mutant rice produced the most significant results when compared to wild Sangyod rice. According to the OSSY-23 results, the rough seed and brown rice sizes are longer at 2.6 mm × 10.1 mm and 2.2 mm × 7.4 mm, respectively.

In addition to this, Pratiwi et al. (2020) study concentrated on Mentik Wangi (local aromatic rice cultivar) originated from Magelang, Central Java, Indonesia. For the Mentik Wangi M2 mutants exposed to 150, 200, and 250 Gy gamma rays, the results showed that 11 mutant plants (150 Gy), 22 mutant plants (200 Gy), and 24 mutant plants (250 Gy) have short stems and high productivity. Destruction of the cell division process and cell expansion as a result of plant height reduction may be related to biochemical and physiological disruption, fluctuation in ascorbic acid content, and auxin loss in plant cells (Hasan et al., 2020).

The next important parameter was the number of tillers which plays an important role in the determination of grain product because it is related to the number of panicles yielded per area (Purwanto et al., 2019). Local rice varieties viz Cempo Melik (100 Gy), Melik (200 Gy) and Cempo Ireng (300 Gy) irradiated with gamma rays each have an average of 25.3, 23.7, and 24.3 tillers, respectively. However, only Cempo Melik irradiated with 100 and 200 Gy gamma rays, and Melik with 100 Gy gamma-ray has 5–10% more productive tillers than the control (Purwanto et al., 2019). Another study by Kurniawati, Chaniago & Suliansyah (2019), found that exposing Sigah brown rice cultivar to 200 Gy gamma-ray increased the number of tillers by 80%, with 99% of the tillers being productive tillers. Although rice plants exposed to gamma rays exhibited short-statured characteristics, the number of tillers and productive tillers increased surprisingly. In addition to these, the number of stem segments and length of each segment are reduced and the plant becomes shorter, which may result in earlier flowering and harvesting. In a study published by Herwibawa, Sakhidin & Dwi Haryanto (2019), aromatic and non-aromatic rice cultivars showed a 70% or greater increase in the number of tillers when exposed to 100 Gy gamma-ray (Inpago Unsoed 1) and 150 Gy gamma rays (Rojolele, Inpari 13 and Cirata) compared to controls. With the incredible increase in the number of tillers, Inpago Unsoed 1 produces 39%, Impari 13 produces 38%, Rojolele produces 64%, and Cirata produces 69% productive tillers from the total number of tillers. As a result of the promising number of productive tillers, Cirata, Inpago Unsoed 1, and Impari 13 produces 71.58 nos, 71.70 nos, and 76.75 number of tillers, respectively, which is much higher than the control.

Plant development is also widely assessed based on the weight of 1,000 grains of rice yield. In Indonesia, Herwibawa, Sakhidin & Dwi Haryanto (2019), Kurniawati, Chaniago & Suliansyah (2019), and Masruroh et al. (2016) have exposed rice to varying doses of gamma rays and discovered an increase in the total weight of 1,000 grains of rice. In Masruroh et al. (2016), for example, Cempo Ireng which was irradiated with 100 Gy gamma-ray produced 12% higher yield, with 11 days early flowering and harvesting periods than the control. While Ciherang was irradiated with 200 Gy of gamma-ray, there was an increase of 4% in the weight of 1,000 grains, with a record of three (3) days early flowering and harvesting periods compared to control. Another study carried out by Kurniawati, Chaniago & Suliansyah (2019) which employed brown rice (Sigah—M3) as a plant material exposed to 200 Gy gamma rays found that 1,000 grains weight was increased by 9% although the flowering and harvesting periods were delayed by 7 and 2, days respectively, compared to the control. Parallel to this, Herwibawa, Sakhidin & Dwi Haryanto (2019) observed that 1,000 grains weight of aromatic upland (Inpago Unsoed 1), aromatic lowland (Rojolele), non-aromatic upland (Cirata), and non-aromatic lowland (Inpari 13) rice cultivars irradiated with 100 and 150 Gy gamma rays were substantially higher than the control. In Malaysia, Yasmine et al. (2019) discovered a 3–8% increase in the total weight of 1,000 grains among Binadhan-8, NMR-152, and Pongsu Seribu-2 rice varieties. As a result, the appropriate dose of physical mutagens based on the variety of rice can stimulate better rice development from vegetative to the mature phase to produce higher yields.

#### Abiotic stress

Tolerance to abiotic stress has also been used to assess rice mutant performance after irradiation. Abiotic stress is defined as the detrimental effect of non-living factors on plants in a specific environment including drought, floods, salinity, extreme temperature, heavy metals, and other natural causes. Herwibawa, Sakhidin & Dwi Haryanto (2019), Amsal & Ishak-Ishak (2018), Yunita et al. (2020), Meesook, Pongtongkam & Poeaim (2018), Che Mohd Zain et al. (2016), and Kadhimi et al. (2016a), for example, have conducted research to identify drought-resistant rice cultivars. Researchers from Malaysia, Che Mohd Zain et al. (2016) and Kadhimi et al. (2016b) exposed rice seeds (MRQ74 and MR269) to 350 Gy of gamma rays and generated osmotic stress using a varied concentration of Polyethylene glycol (PEG) 6,000 ranging from 0–20%. The results of both types of research revealed that the highest proline concentrations of 29.156 and 40 µMg^−1^ was noticed at PEG concentration levels of 7% and 20%, respectively, under *in vitro* condition. Meanwhile, in Thailand, Meesook, Pongtongkam & Poeaim (2018) irradiated riceberry rice seeds with gamma rays at 0, 20, 25, 30, 35, and 40 krad; however, the acceptable survival rate was found to be lower for those above 28 krad, thus, the proline concentrations of leaf were evaluated between 0 to 28 krad. The data showed that proline concentrations were higher in the radiation treatment at 15% PEG (0.175 µMg^−1^) compared to non-irradiate (0.15 µMg^−1^) at the same concentration PEG. In Indonesia, rice seeds (Inpago Unsoed 1, Rojolele, Inpari 13, and Cirata) were irradiated with 100 or 150 Gy gamma rays were immersed in sodium azide (SA) for two (2) or six (6) h before being treated with 5% PEG at a pressure of −0.03 MPa. Under the drought stress level of −0.03 MPa, the genetic variants in the M1 generation show diversity, and the best combination of mutagen and cultivar is Inpago Unsoed 1, which was irradiated with gamma-ray 100 Gy and then soaked in SA for 2 h (Herwibawa, Sakhidin & Dwi Haryanto, 2019). Amsal & Ishak-Ishak (2018) exposed the Mira-1 rice variety to 25 and 50 Gy of gamma rays and tested with 20% PEG resulting in two (2) from 20 mutant lines; 28H1 and 30F1 mutant lines showing the best characteristics (number of productive tillers; 12 each, lateral root; 142 and 100, and grain yield per panicle; 80 and 124, respectively) for drought tolerant. Lateral roots can develop on any primary root, including crown roots and embryonic, and are used as a drought tolerance indicator. The ability of the 28H1 and 30F1 rice mutants to absorb water in dry conditions may benefit from having the greatest number of lateral roots. Callus mutation with gamma rays was used by Yunita et al. (2020) in upland rice (Situpatengang and Batutegi) where Situpatengang was irradiated with 24.68 Gy gamma rays and Batutegi was with 22.15 Gy gamma rays. Both varieties were tested with 24.11% and 25.18% PEG, respectively, and both mutant calluses were regenerated on MS +BA 3 mg/L + Zeatin 0.1 mg/L, produced 83 shoots Situpatengang and 73 shoots in Batutegi callus. After acclimatization, the shoots produced 52 mutant lines from Situpatengang rice varieties and 49 mutant lines were produced from Batutegi rice varieties.

Malumpong et al. (2019) used fast neutrons on rice to cope with salt stress during the reproductive stage, and Promnart et al. (2017) used electron beams to explore flood tolerance in Thai rice breeds. A total of 9,000 lines of irradiated M_4_ JHN with 33 Gy fast neutron were screened for salt tolerance (150 mM NaCl) and seeded in a field nursery, with M1145 emerging as the salinity tolerant line. Even though the lines were similar under salt stress to Pokkali and FL530 (typical salt-tolerant varieties), the researcher proposed that M1145 should be evaluated in the field to confirm the potential for salt stress. Furthermore, in the flood tolerance study, approximately 3,000 M_3_ lines of irradiated electron-ion beam 0.44 KGy were screened for submergence tolerance and 317 tolerant mutant lines were survived and selected for planting for the following season (M_4_ and M_5_).

A combination of the above study results reported to the impact of irradiation on abiotic stress is expected to be used as a benchmark, particularly in drought, flood, and salinity, in designing the upcoming abiotic resistant/tolerant rice genetic line.

#### Biochemical properties

Radiation may also alter the gene that regulates biochemical compounds in plants. Anthocyanin, the bioactive substance with antioxidant properties in rice that may give anti-inflammatory and anti-cancer benefits, is one of the primary target qualities of the next generation of genetically modified crops particularly in black rice (Chen & Lin, 2013; Ciulu, de la Cadiz-Gurrea & Segura-Carretero, 2018). According to Yamuangmorn & Prom-U-thai (2021), anthocyanin can reduce cell damage when plants are exposed to biotic and abiotic stress, and anthocyanin’s contribution to plant structure has been identified as protecting against free radicals during physiological metabolism. Generally, the subspecies indica and japonica were reported to have total anthocyanin contents ranging from 32.40 to 50.30 mg/100 g, and 121.30 to 155.90 mg/100 g, respectively (Pedro, Granato & Rosso, 2016). However, according to Masruroh et al. (2016), Cempo Ireng rice exposed to 500 Gy of gamma-ray resulted in a 52.174% increase in anthocyanin levels compared to controls (18.623%). A similar study conducted by Purwanto et al. (2019) discovered the highest concentration of anthocyanin in Cempo Ireng rice irradiated at 200 Gy of gamma-ray with 155.946 mg/100 g and Cempo Melik with 142.492 mg/100 g (300 Gy of gamma-ray). In contrast, Melik rice had lower anthocyanin contents with 137.523 mg/100 g (100 Gy of gamma-ray) compared to control 146.323 mg/100 g as the radiation exposure increased (Purwanto et al., 2019). This suggests that radiation has a strong effect on important biochemical contents of rice and may contribute to enhancing the property at the right dosage.

#### Nutritional quality

Higher nutritional value food is constantly required for human health. Agricultural practices (soil preparation, sowing, manuring, irrigation, *etc*.), post-harvest handling (threshing, drying, cleaning, milling, grading, storage, and packaging), cultivar varieties and manipulations, followed by selection through breeding and genetic mechanisms, all have an impact on the nutrient content of rice. It is necessary to carefully manage the entire process to avoid nutritional losses including in the selection of grains for seeds to be used in the following cropping. The current study provides an update on the effect of physical mutagens on the rice’s nutritional properties.

The protein content is one of the nutrients that represents the nutritional quality of the rice yield. According to Amagliani et al. (2017), the protein content of rice grain varies and can be classified as brown rice, white or milled rice, and rice bran. The protein content of each type of rice grain ranged from 7.1% to 8.3% (brown rice), 6.3% to 7.1% (milled rice), and 11% to 15% (rice bran). Current reviews have found that Masruroh et al. (2016) have investigated the effects of five (5) levels of gamma radiation (100, 200, 300, 400, and 500 Gy) on two (2) rice cultivars to improve rice nutritional quality of Ciherang and Cempo Ireng. As a result, the protein content of Ciherang rice exposed to 400 Gy gamma rays increased by 43%, with a protein content of 8.98% compared to the control, which was 6.26%. In comparison to the control (7.73%), Cempo Ireng exposed to 500 Gy gamma-ray increased by 30%, with a protein content of 10.09%. Another study by Techarang et al. (2018) on protein content in brown rice (M2 generation) from RD6 mutants exposed to ion beams (4 × 10^16^ ions/cm^2^ (50 kV)) found that OSRD6-4 and OSRD6-20 are 9.1/100 and 8.7/100 g, respectively, and are higher than the wild RD6 (8.3/100 g). Similarly, OSSY-23 (8.9/100 g) and OSSY-17 (9.3/100 g) were mutants selected from Sangyod rice mutants in M2 generation irradiated with an ion beam (4 × 10^16^ ions/cm^2^ (50 kV)) also showed high protein content compared to wild Sangyod (8.6/100 g). The findings demonstrated that when physical agents are used in the induced mutation breeding technique, rice characteristics have a high possibility and chance of improving rice quality.

Amylose content was the next characteristic that represents nutritional quality in rice. The amylose content of the milled rice is a major determinant of rice texture, specifically rice hardness, and this contributes to amylose content playing an important part in decision making in rice breeding programs. Amylose content in rice is divided into five (5) categories: waxy (0–2%), very low (3–9%), low (10–19%) intermediate (20–25%), and high (>25%) (Cuevas et al., 2016). Techarang et al. (2018) discovered two (2) RD6 rice mutants (M2 generation), OSRD6-4 and OSRD6-20, that showed beneficial changes in amylose content after being exposed to an ion beam (4 × 10^16^ ions/cm^2^ (50 kV)). The amylose content of the M2 generation of RD6 rice which is OSRD6-4 (16.4/100 g) was higher than that of the RD6 (wild type) with amylose content of 7.0/100 g and OSRD6-20 (6.8/100 g). Meanwhile, M2 generation from Sangyod rice revealed no significant difference in amylose content between rice mutants OSSY-14 (19.0/100 g), OSSY-17 (15.5/100 g), and OSSY-23 (16.9/100 g) compared to wild Sangyod (17.7/100 g) when exposed to ion beam (4 × 10^16^ ions/cm^2^ (50 kV)). This implies that wild rice Sangyod, Sangyod mutants (OSSY-14, OSSY-17, and OSSY-23), and OSRD6-4 belong to the low amylose content group, whereas wild RD6 and RD6 mutants (OSRD6-20) belong to the group very low amylose content. When cooked, rice with a very low amylose content becomes softer, moister, and stickier in texture than rice with low amylose content. Furthermore, according to Prom-u-thai & Rerkasem (2020), grain with a higher amylose content absorbs more water, resulting in a higher volume expansion ratio, such as the volume of cooked rice compared to its precooked volume. Based on the findings, it is possible to conclude that using the optimum physical mutagens such as gamma rays and ion beams may result in a wide range of morphological changes, including amylose content (glutinous to non-glutinous) and protein content.

#### Industrial quality

There is currently no common definition of “industrial rice quality,” and there is even less agreement on how it should be quantified. Rice quality was difficult to define precisely because “rice quality” is subjective and context-specific compared to agronomic traits which include the ability of rice to boost production and/or alleviate abiotic or biotic stresses in crops (Custodio et al., 2019). Consumer perceptions of rice quality differ between areas, countries, cities, and levels of urbanization. According to Jaafar, Lalp & Mohamed@Naba (2018), consumer attitudes, and extrinsic and intrinsic attributes were factors that influence consumers’ purchase intention toward food products in Malaysia. In this study, rice quality characteristics such as grain shape, size, color, cleanliness, softness, and aroma can be categorized as intrinsic elements, whereas extrinsic elements include price, labeling, packaging, and branding. The intrinsic quality elements could also apply to rice-based products such as rice noodles.

In Ahmad Zainuri et al. (2020), gamma irradiation (4, 6, and 8 KGy) on flat rice noodles was found to cause changes in physicochemical properties such as texture, color, and cooking quality. Increasing the gamma-ray dosage, decreased the pH value, lightness (dark to white), cooking yield (%) (weight of noodle after cooked/weight of uncooked noodle), and hardness of the flat rice noodle. However, this increases cooking loss (noodle particles leaked into the cooking water), breaking length and color changes. Parallel to this, it was found that 4 KGy of gamma-ray lowers microbial growth and 8 KGy helps extends up to ten (10) days of shelf life of flat rice noodles at room temperature. As a consequence, gamma rays not only promote growth and yield, respond to abiotic stress, and improve the biochemical properties and nutritional values, but it also improves a product’s industrial quality and potentially extend storage duration.

Semsang et al. (2018) employed low energy N-ion beams in addition to gamma rays to improve the industrial quality of rice. The introduction of a low-energy ion beam as a source of irradiation comes with characteristics such as low damage rate, high mutation rate, and broad mutation spectrum. Bearing this in mind, Semsang et al. (2018) conducted a study focused on seed quality losses that occur throughout the growing season, harvesting, and storage as the primary goals by using ion beam mutation in Thailand. The results showed that HyKOS21 (a secondary mutant of KDML 105) derived from the Thai jasmine rice variety, KDML 105 (as a wild type), and BKOS6 (primary mutant of KDML 10) have favorable traits such as short stature, photoperiod insensitivity, and high yield potential. Not just that, HyKOS21 lacks the expression of the LOX-1 and LOX-2 genes, indicating that it increases the life span of seeds. This is evidenced by the fact that the germination percentage of seeds stored in natural conditions can reach up to 90% on average after 18 months of storage, compared to less than 50% for wild type.

## Future prospective

The use of physical mutagens in rice breeding is a promising tool for improving rice production and addressing rice cultivation challenges. Previously, this approach may have aided in the selection and evaluation of desirable traits such as improved yield, drought stress tolerance, and disease resistance by exposing rice seeds to ionizing radiation to induce genetic mutations.

To accelerate the development and deployment of improved rice varieties in the future, it is advisable to combine radiation-induced mutation breeding with modern molecular and genetic techniques, such as gene editing and marker-assisted selection. Furthermore, as demonstrated in the current study, gamma rays were the most widely used as a mutagen agent, owing to the high cost and the need for specialized facilities of using neutrons as a mutagen. It is crucial to develop new irradiation methods that are more cost-effective and efficient such as using low-dose or targeted radiation exposure. In addition, current research is primarily focused on agronomic characteristics, but there is a gap in the nutritional quality of rice through radiation-induced mutation breeding, which involves selection of mutations that increase the levels of essential nutrients such as iron and vitamin A. To address the scarcity of arable land, it is crucial to increase the sustainability of rice production by developing new rice varieties that require less water, fertilizer, and pesticides and that are more resistant to pests and diseases. Rice remains an important food crop in many countries and thus continuing to advance the understanding of induced mutation and its application will help in making the supply enough for all.

## Conclusions

Handful integrated research on the impacts of chemical mutagens on rice has been conducted in recent years, with only a few related to physical mutagens. As a result, the current works emphasize the need for additional research on the use of physical mutagens in rice breeding, particularly in Southeast Asia. Overall, the database shows that gamma rays exhibit physical mutagens that are commonly utilized in rice variety enhancement from 2016 to 2020.

To achieve better rice production traits (plant development, biochemical properties, abiotic stress, nutritional quality, and industry quality), gamma-rays have played a significant role in plant genetics studies with Cesium-137 and Cobalt-60 as a radiation source. While the ideal dosage rate is between 100 to 250 Gy to produce a more stable plant character and lower the chromosomal damage and less negative side effects. In addition, it was found that seeds were widely used as a source of plant materials in physical mutagen research. The findings from this review are useful as a guideline to determine the correct irradiation dosage and the benefits from this usage of physical mutagens that might contribute to the enhancement of global food security.

## Additional Information and Declarations

### Data Availability

The following information was supplied regarding data availability: This is a literature review.
